# Using genetically encoded fluorescent biosensors to interrogate ovarian cancer metabolism

**DOI:** 10.1186/s13048-022-01046-5

**Published:** 2022-10-20

**Authors:** Shree Bose, Haipei Yao, Qiang Huang, Regina Whitaker, Christopher D. Kontos, Rebecca A. Previs, Xiling Shen

**Affiliations:** 1 Department of Biomedical Engineering, Duke University Pratt School of Engineering, Durham, NC USA; 2 Department of Pharmacology and Cancer Biology, Duke University School of Medicine, Durham, NC USA; 3 Department of Pediatric Surgery, Second Affiliated Hospital of Xi’an Jiaotong University, Xi’an, Shaanxi China; 4 Department of Obstetrics and Gynecology, Division of Gynecologic Oncology, Duke University Medical Center, Durham, NC USA; 5 Department of Medicine, Division of Cardiology, Duke University Medical Center, Durham, NC USA; 6 Terasaki Institute for Biomedical Innovation, Los Angeles, CA USA

## Abstract

**Background:**

Epithelial ovarian cancer (OC) is the most lethal gynecological malignancy and patients present with significant metastatic burden, particularly to the adipose-rich microenvironment of the omentum. Recent evidence has highlighted the importance of metabolic adaptations in enabling this metastasis, leading to significant interest in evolving the arsenal of tools used to study OC metabolism. In this study, we demonstrate the capability of genetically encoded fluorescent biosensors to study OC, with a focus on 3D organoid models that better recapitulate in vivo tumor microenvironments.

**Materials and methods:**

Plasmids encoding the metabolic biosensors HyPer, iNap, Peredox, and Perceval were transfected into 15 ovarian cancer cell lines to assay oxidative stress, NADPH/NADP+, NADH/NAD+, and ATP/ADP, respectively. Fluorescence readings were used to assay dynamic metabolic responses to omental conditioned media (OCM) and 100 μM carboplatin treatment. SKOV3 cells expressing HyPer were imaged as 2D monolayers, 3D organoids, and as in vivo metastases via an intravital omental window. We further established organoids from ascites collected from Stage III/IV OC patients with carboplatin-resistant or carboplatin-sensitive tumors (*n* = 8 total). These patient-derived organoids (PDOs) were engineered to express HyPer, and metabolic readings of oxidative stress were performed during treatment with 100 μM carboplatin.

**Results:**

Exposure to OCM or carboplatin induced heterogenous metabolic changes in 15 OC cell lines, as measured using metabolic sensors. Oxidative stress of in vivo omental metastases, measured via intravital imaging of metastasizing SKOV3-HyPer cells, was more closely recapitulated by SKOV3-HyPer organoids than by 2D monolayers. Finally, carboplatin treatment of HyPer-expressing PDOs induced higher oxidative stress in organoids derived from carboplatin-resistant patients than from those derived from carboplatin-sensitive patients.

**Conclusions:**

Our study showed that biosensors provide a useful method of studying dynamic metabolic changes in preclinical models of OC, including 3D organoids and intravital imaging. As 3D models of OC continue to evolve, the repertoire of biosensors will likely serve as valuable tools to probe the metabolic changes of clinical importance in OC.

**Supplementary Information:**

The online version contains supplementary material available at 10.1186/s13048-022-01046-5.

## Introduction

Epithelial ovarian cancer (OC) remains the most lethal gynecological cancer, with ~ 300 K new cases and ~ 200 K deaths reported worldwide in 2020 [1]. The causal factors that underlie the high rate of OC mortality are multifold. Due to a lack of effective early screening methods and minimal or no symptoms at an early stage, patients with OC are often diagnosed after tumors have already metastasized throughout the peritoneum. In particular, OC has been shown to preferentially metastasize to and rapidly proliferate in the omentum, the fat pad that overlies the abdominal cavity. For patients that present with advanced stage disease, cytoreductive surgery and platinum-based chemotherapy are the standard of care. However, these treatment modalities are frequently ineffective, with a majority of patients ultimately experiencing recurrent disease with high rates of drug resistance [2]. Thus, the clinical challenges of metastatic disease and chemoresistance have remained the major hurdles to improving morbidity and mortality for patients with OC. As such, further investigations to better understand and target the factors that underlie these phenomena are warranted.

The precise cellular mechanisms responsible for the aggressive nature of OC omental metastases are not well understood; however, recent evidence has underscored the importance of metabolic reprogramming in facilitating OC omental metastases. Nieman et al. showed that OC cells co-cultured with adipocytes adopted lipid uptake and fatty acid (FA) β-oxidation to support rapid proliferation [3]. Evidence supporting this metabolic interplay between adipocytes and metastatic OC cells comes from studies demonstrating that adipocytes secrete adipokines like monocyte chemoattractant protein-1 (MCP-1), interleukin-6 (IL-6), IL-8, tissue inhibitor of metalloproteinase-1 (TIMP-1), and adiponectin [4]. Furthermore, metastasizing OC cells have been shown to upregulate expression of the lipid chaperone protein fatty acid binding protein 4 (FABP4), particularly in cells at the adipocyte-tumor interface and FA receptor, CD36 [5, 6]. Other key regulators of cell metabolism, including salt-inducible kinase 2 (SIK2), have also been implicated as important drivers of global metabolic changes in omental OC metastases [7, 8]. Using discrepancies in gene expression data from human ovarian and omental tumors as the foundational hypothesis for further work in preclinical models, these studies have established the importance of metabolism in OC pathophysiology.

In recent years, cancer models have expanded to include the use of 3D organoid cultures as robust tools to recapitulate tumor genetic and epigenetic diversity, drug responses, and intercellular dynamics. In OC, biobanks of patient-derived organoids (PDOs) have been shown to capture intra- and interpatient heterogeneity and drug responses [9, 10]. CRISPR-Cas9 genome editing tools have been applied to selectively mutate murine oviductal organoids to interrogate OC carcinogenesis [11]. Genomic and functional testing of OC PDOs has revealed that DNA damage repair defects can be targeted using therapeutic agents [12]. However, despite this evidence suggesting that organoids can faithfully recapitulate patient tumor behavior, metabolism in OC organoids remains largely unexplored.

Mass spectrometry, enzymatic cycling assays, and flux measurement methods have emerged as standard techniques to study metabolic states. However, each of these methods rely on sample destruction, therefore they provide only static measurements at a given time point. In addition, technical challenges in implementing these methods in the complex cultures of 3D organoids have stymied extensive investigation of organoid metabolism. The application of optical metabolic imaging (OMI) to intestinal stem cells and some cancer organoids has yielded some insights [13–15]. For example, a study that performed OMI on breast and pancreatic cancer organoids found that metabolic changes could predict patient responses to chemotherapy [16]. However, as FLIM requires highly specialized equipment and remains limited to measuring only endogenous redox states, more tools will be necessary to study metabolism in organoids as preclinical models of disease.

Recently, a novel class of genetically encoded fluorescent biosensors has expanded our ability to interrogate real-time metabolic changes using live-cell imaging. These sensors present an efficient means to study dynamic metabolic states, as they can be easily introduced into cells via transfection, electroporation, or infection, and subcellular localization can be achieved using intracellular targeting sequences [17]. The rational design of many of these sensors, joining metabolite-sensing regions of endogenous proteins with fluorescent proteins, also allows a dynamic range through the use of various fluorescent reporters. Factors that affect the fluorescence signal, e.g., variable rates of expression, pH-sensitivity, etc., can be controlled for using ratiometric designs and pH-resistance. With an arsenal of sensors that is steadily growing, interrogation of many cellular states is now possible, including NADPH/NADP+ (iNap sensors [18]), NADH, NAD+ (Peredox [19] and SoNar [20] sensors), and ATP/ADP ratios (Perceval sensor [21]). Moreover, modification of these biosensors supports analysis of metabolic perturbations. HyPer-DAAO, a H_2_O_2_ reporter joined with a D-amino acid oxidase (DAAO), allows selective induction of intracellular oxidative stress when cells are exposed to physiologically inactive D-alanine [22].

Here, we applied these biosensors to interrogate ovarian cancer metabolism, particularly in the context of 3D model systems, and found that the resulting metabolic readouts could provide a useful method of studying dynamic metabolic changes in preclinical models and patient-derived organoid models of OC.

## Materials and methods

### Patient studies

Patients with advanced stage and/or recurrent high grade serous ovarian cancer (HGSOC) were classified as either “responders” or “non-responders” based on response to platinum-based chemotherapy (*n* = 4 per group). Human malignant ascites samples were obtained from the Duke University School of Medicine Ovarian Cancer Research Biobank under protocol ID Pro00013710 (Banking Normal and Malignant Gynecologic Tissues Removed at Surgery). Ascites samples were obtained prior to tumor resection or chemotherapy treatment and were centrifuged to isolate the cellular fraction. Ascitic fluid cell pellets were stored in FBS with 10% DMSO and were thawed prior to organoid development.

The ascitic pellet was resuspended in ice-cold HBSS and centrifuged at 1000 rpm for 5 minutes. Wash was performed twice, and then cells were counted and normalized to have 1000 cells/20 uL Cultrex Basement Membrane Extract (RND Systems) droplet. For samples in which significant cellular aggregates were noted, TrypLE was added, and samples were incubated for 5 minutes at 37 °C. Cells were spun down once more and resuspended in Matrigel before carefully plating 25 μM droplets on pre-warmed 24 well plate. The plate was then put in the 37 °C incubator for 5 minutes to allow the matrix to solidify, before media was gently added on top. Composition of organoid media is as noted in Supplementary Table 2.

The HyPer-DAAO construct was transfected into PDOs using Lipofectamine 2000 according to manufacturer’s instructions, and expression was confirmed using a fluorescence microscope (Olympus OV100). PDOs were treated with 100 μM carboplatin as previously described [23] and HyPer response was evaluated 48 hours post-treatment by emission measurements at 500 nm following excitation at 420 nm and 480 nm using a Varioskan plate reader.

### Mouse studies

Athymic, nude (Foxn1^nu^) female mice, 6–8 weeks old were obtained from the Jackson Laboratory and maintained in compliance with Institutional Animal Care and Use Committee (IACUC) guidelines. Mice were injected with 1 × 10^6^ SKOV3-HyPer cells intraperitoneally for intravital imaging or subcutaneously for organoid development.

At the endpoint of 14 days, mice were euthanized by CO_2_ inhalation and tumors were harvested for further organoid culture. The time from dissection to organoid plating (i.e., cold ischemic time) was limited was < 1 hr.

### Intravital imaging

For intravital imaging, animals were anesthetized with isoflurane (4% vol/vol induction with 2–3% maintenance). Mice were injected with bupivacaine (0.25% < 2 mg/kg) and meloxicam (2 mg/kg once) subcutaneously prior to surgery. Surgical site was cleaned by alternating iodine and 70% ethanol treatments. A 15-mm diameter circle of the abdominal skin was surgically removed. The omentum was gently brought to the surface of the abdomen and the titanium window with a glass insert was placed on top. The window was then sutured to the abdominal wall on either side of the window and Loctite 406 (Ellsworth, 135,436) was used to adhere to the surrounding skin. SKOV3-HyPer cells were injected 24 hours after intravital window placement, and imaging of omental OC cells was performed using two-photon microscopy (Leica SP8 2-Photon DIVE) at 72 hours.

### SKOV3-HyPer organoid establishment

Organoids were established according to previously described methods [10]. In brief, following isolation, tumor tissue was stored in AdDF+++ (Advanced DMEM/F12 containing 1× Glutamax, 10 mM HEPES and antibiotics), prior to being washed 3x in ice-cold PBS. Tissue was then minced finely in a small amount of HBSS and centrifuged to form a cell pellet. Digestion was carried out using AdDF+++ supplemented with 5 μM RHO/ROCK pathway inhibitor (Abmole Bioscience, Y-27632) containing 0.5–1.0 mg/mL collagenase (Sigma, C9407) at 37 °C for 1 hour, after which cells were spun down. The remaining cell pellet was resuspended in Matrigel (Corning Sciences, Inc.) and plated in 25 uL domes in 24 well plate. Organoid culture media composition is shown in Supplementary Table 2.

### OC cell line culture

Fifteen ovarian cancer cell lines were obtained as a gift from the Duke Gynecological Oncology Research Group. These cell lines are shown in Fig. 2. All ovarian cancer cell lines were cultured in DMEM medium supplemented with 10% fetal bovine serum (FBS) (Corning Life Sciences) and 1% penicillin/streptomycin/L-glutamine (Corning Life Sciences).

To generate the omental conditioned media for culture, fresh omenta were isolated from sacrificed C57BL/6 J mice. After 3 washes in PBS to remove residual clots from dissection, omenta were minced and cultured in DMEM without FBS for 48 hours. The adipose tissue was then removed, and the media was passed through a 45 μM filter prior to use in cell culture applications.

Carboplatin (Sigma-Aldrich) was dissolved in water and aliquoted to stock solutions of 15 mM kept at − 20 °C, made fresh prior to cell culture treatment and protected from light. The carboplatin dosage was 100 μM for all experiments.

### Constructs

Constructs for HyPer-DAAO (Thomas Michel, Addgene), iNAP (Yiping Yi, gift), Perceval (Gary Yellen, Addgene), and Peredox (Gary Yellen, Addgene) biosensors were obtained.

For 2D cultures, constructs for HyPer-DAAO, iNap, Perceval, and Peredox were transfected using Lipofectamine 2000 according to manufacturer protocols and were maintained in DMEM + 10% FBS until fluorescent expression was seen under a fluorescence microscope (Olympus OV100). Cells were then treated with OCM or carboplatin, and biosensor signals were quantified by fluorescent excitation and readings at the emissions at the wavelengths shown in Table 1 (Varioskan plate reader).

**Table 1:** Biosensors used for this investigation

Metabolic State	Sensor	Excitation/Emission	Sensing Domain	Source
Intracellular H_2_O_2_	HyPer-DAAO	420/500 and 480/500 nm	OxyR, H_2_O_2_ sensor/transcription factor *(Escherichia Coli)*	[24]
NADPH:NADP+	iNap	420/528 and 485/528 nm	NAD(H) binding domain of Rex *(Thermus aquaticus)*	[18]
ATP:ADP	Perceval	405/530 and 490/530 nm	ATP-sensing domain from GlnK1 *(Methanococcus jannaschii)*	[21]
NADH:NAD+	Peredox	405/525 and 587/629 nm	NAD(H) binding domain of Rex *(Thermus aquaticus)*	[19]

To generate stably expressing SKOV3-HyPer cells, HEK293T cells were transfected with psPAX2 and pMDG.2 packaging plasmids and the HyPer-DAAO construct cloned into a lentiviral vector backbone. Viral supernatant was collected after 48 and 72 hours and concentrated using LentiX concentrator (Takara Bio). SKOV3 cells were infected and stably expressing cells were selected using FACS.

### Statistics

Statistical analysis was performed using R (https://cran.r-project.org/). Parametric data were calculated by Students T-tests. All correlation analyses were performed with Pearson’s correlation coefficient. In all analyses, a two-sided *P* value< 0.05 was considered statistically significant. Figures were drawn using GraphPad Prism 8.0 software (San Diego, USA).

## Results

### Temporal tracking of metabolic responses to Omental conditioned media

**Fig. 1:**
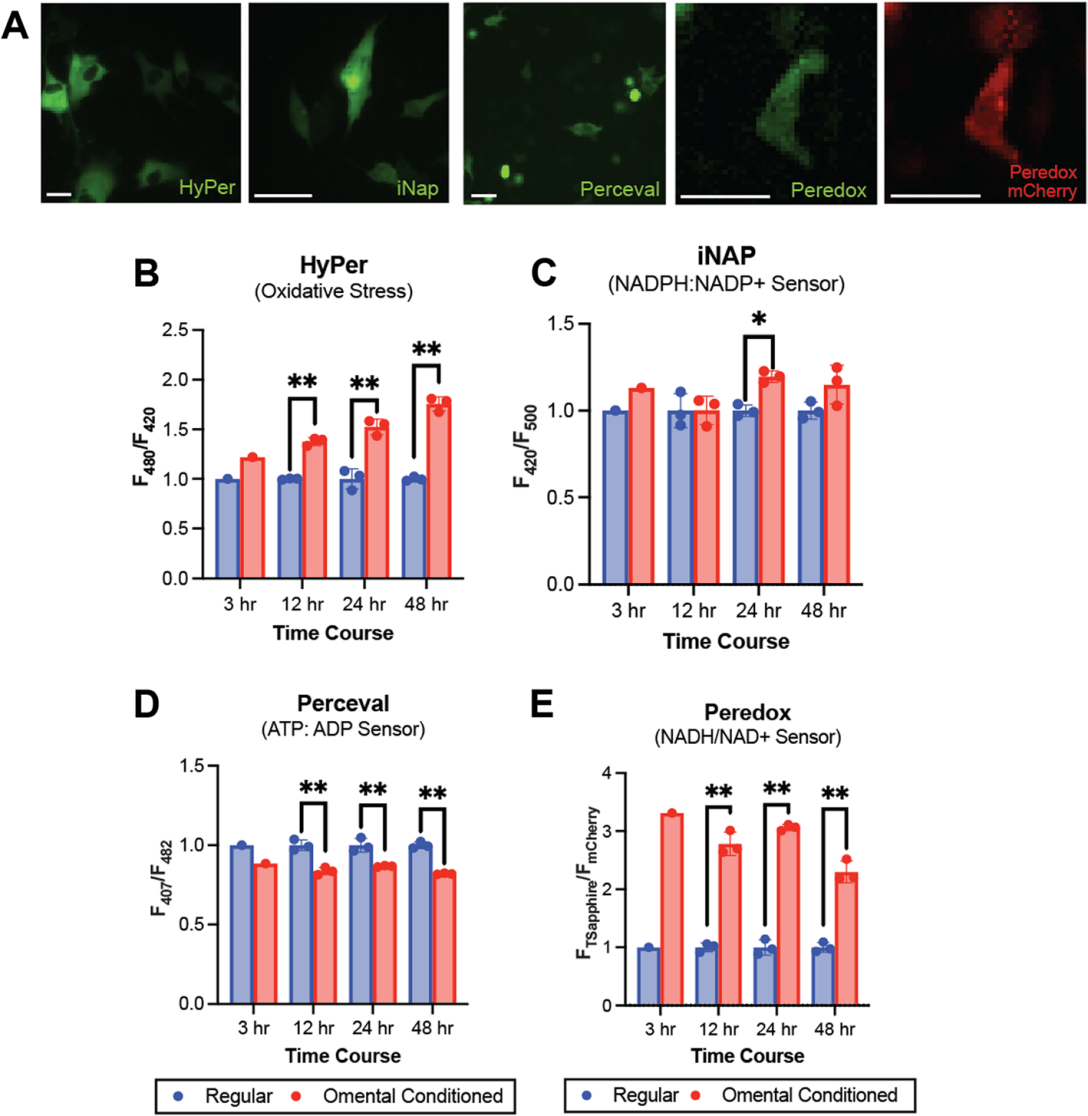
Genetically encoded, fluorescent biosensors allow tracking of dynamic changes in OC metabolism. **A** Representative fluorescence micrographs verifying expression of HyPer, iNap, Perceval, and Peredox biosensors. Quantification of HyPer (**B**), iNap (**C**), Perceval (**D**), and Peredox (**E**) signals was performed on transfected SKOV3 cells grown in regular media vs. omental conditioned media (OCM) for 3, 12, 24, and 48 hours. Signals were normalized to regular media controls (*n* = 3, technical replicates). Statistical significance was calculated using an unpaired, two-tailed Students *t*-test (*p < 0.05, **p < 0.01)

Given the clinical relevance of OC behavior in the omentum, we first investigated the metabolic responses of OC cells exposed to omental conditioned media (OCM) over time. After transfecting cohorts of SKOV3 OC cells with plasmid constructs for iNap, HyPer, Perceval, or Peredox, biosensor expression was verified using fluorescence microscopy (Fig. 1A). Transfection efficiency of ~ 80% was achieved for all constructs (not shown). These cells were then exposed to OCM and compared to control cells grown concurrently in regular media. Measurements at 3, 12, 24, and 48 hours following treatment revealed differences in metabolic states. HyPer measurements of intracellular H_2_O_2_ revealed increasing oxidative stress over time with growth in OCM (Fig. 1B), whereas iNap measurements revealed a modest but significant increase in NADPH/NADP+ ratio at 24 hours that appeared to be sustained at 48 hours, although not significant (Fig. 1C). In contrast, measurements of Perceval, a sensor that utilizes competition between ATP and ADP for the GlnK1 active site to reliably report the ATP/ADP ratio, demonstrated consistent, decreased ATP/ADP ratios for OCM-treated SKOV3 cells at all time points (Fig. 1D). Conversely, NADH/NAD+ ratios were consistently increased with OCM exposure across all time points, as measured using Peredox (Fig. 1E). Peredox is not a ratiometric sensor but rather includes an NADH/NAD+ sensing domain with a circularly permuted T-Sapphire fluorescent protein (FP) conjugated to mCherry, which serves as the internal control. Expression of both FPs were validated prior to OCM assays (Fig. 1A).

### OC cell lines demonstrate heterogeneity in metabolic responses to OCM treatment

**Fig. 2:**
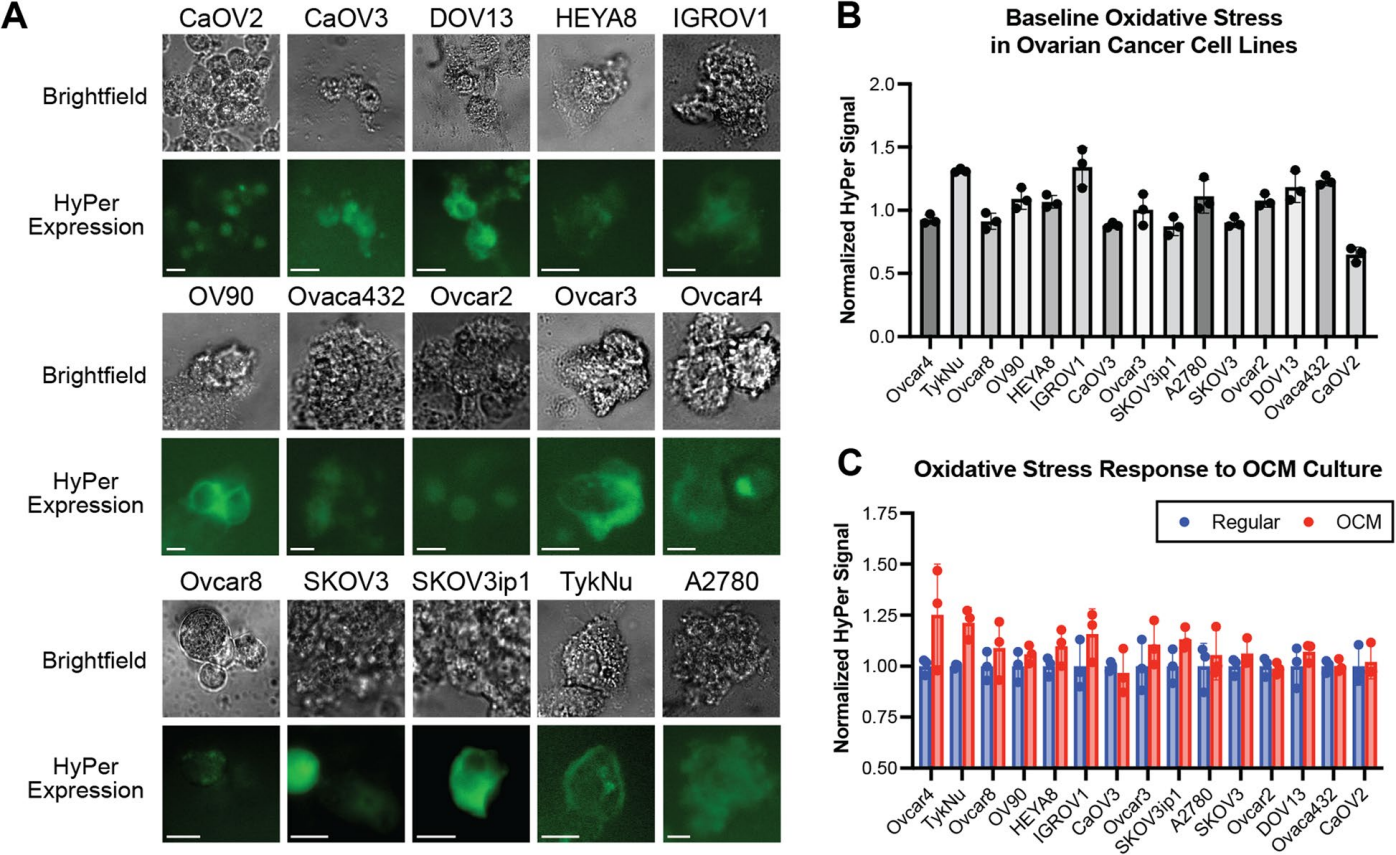
Different OC cell lines display heterogeneous biosensor responses at baseline and in response to OCM. **A** Baseline oxidative stress was evaluated in 15 OC cell lines transfected with HyPer-DAAO and HyPer. Scale bars indicate 100 um. **B** Oxidative stress in HyPer-expressing cell lines was measured using fluorimetry (excitation at 420 and 480 nm) and a comparative analysis was performed. A baseline average of measurements across cell lines was used for normalization. **C** HyPer signal was quantified after 48 hours of growth in OCM or regular media and normalized to control, regular media-treated cells

To characterize the intratumoral and interpatient heterogeneity that contributes to the diverse nature of OC, we next investigated metabolic responses across a panel of human OC cell lines using these sensors. Cultures of 15 different OC cell lines were transfected with the HyPer-DAAO construct to induce oxidative stress (Fig. 2A). Responses to this intracellular oxidative stress, measured using HyPer, varied among OC cell lines at baseline (Fig. 2B). Similarly, the response to OCM treatment, normalized to baseline measurements for each cell line, was also highly variable (Fig. 2C). CaOV2, a cell line derived from malignant ascites, exhibited the lowest baseline oxidative stress compared to other cell lines. Interestingly, treatment of CaOV2 with OCM did not result in a significant increase in oxidative stress for this cell line, unlike other cell lines, like TykNu, a primary tumor-derived cell line (Fig. 2C). Additional analyses of these 15 cell lines were carried out using iNap, Perceval, and Peredox (Supplementary Fig. 1). As with oxidative stress responses, similar heterogeneous responses were observed across the OC cell lines for each of the other metabolic biosensor measurements.

### Metabolic changes induced by carboplatin treatment

**Fig. 3:**
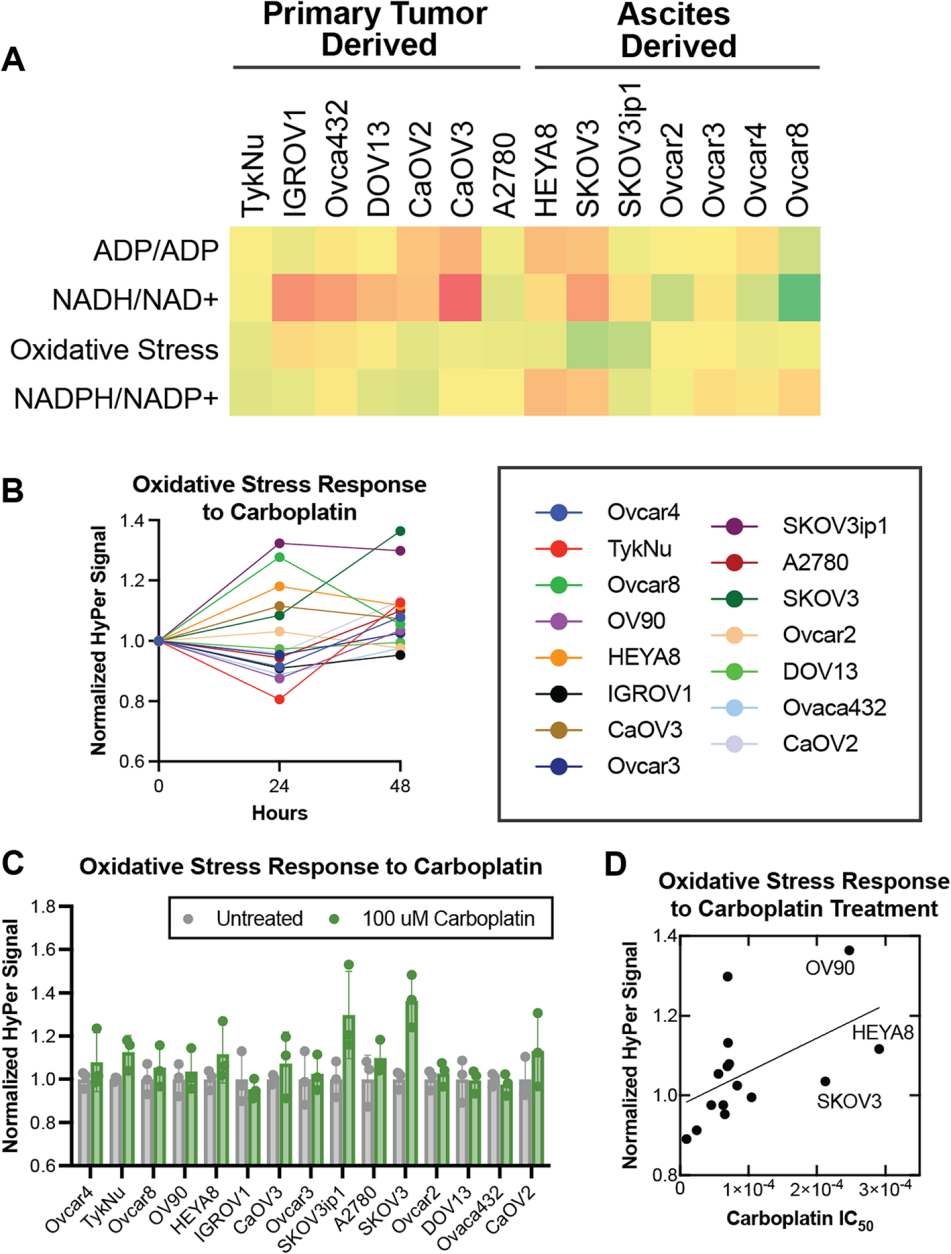
OC cell responses to carboplatin-induced oxidative stress are correlated with carboplatin resistance. **A** Metabolic changes in ATP/ADP, NADH/NAD+, oxidative stress, and NADPH/NADP+ induced by treatment with carboplatin varied across cell lines derived from primary tumor vs. ascites. Color bars represent the degree of change (green = greater change, red = minimal change from baseline). **B** HyPer-expressing cell lines were treated with 100 μM carboplatin and HyPer signal was measured at 24 and 48 hours. **C** HyPer responses in carboplatin-treated cells were compared to untreated cells normalized to 1 (*n* = 3 per condition). **D** Normalized HyPer responses for each cell line were plotted against reported carboplatin IC50s, and a simple linear regression demonstrated a positive correlation (*r* = 0.53, *p* = 0.041)

Given the importance of platinum-based chemotherapy as the first-line treatment for OC, we sought to characterize heterogeneity in the metabolic response to carboplatin. Significant heterogeneity in NADH/NAD+, ATP/ADP, NADPH/NADP+, and oxidative stress induced by treatment with carboplatin was measured in 15 primary tumor and ascites-derived OC cell lines (Fig. 3A). HyPer signal was measured in HyPer-expressing cell lines 24 and 48 hours after treatment with 100 μM carboplatin (Fig. 3B). At 48 hours, oxidative stress was generally increased, with the most pronounced increase from baseline in SKOV3 cells (Fig. 3C). Visual inspection of cells revealed that biosensor fluorescence was not observed in dead cells (not shown), consistent with previously published studies [25].

We next sought to determine whether carboplatin-induced oxidative stress is related to carboplatin resistance in individual cell lines. Because there is appreciable resistance to platinum-based first-line therapies in OC, carboplatin has been extensively studied in OC preclinical models, including the cell lines used in this study. Carboplatin IC50s previously reported in the literature (Supplementary Table 1) were compared to observed oxidative stress responses induced by 100 μM carboplatin treatment in the 15 cell lines (Fig. 3D). Cell lines with greater carboplatin resistance (higher carboplatin IC50) generally exhibited greater carboplatin-induced oxidative stress responses with a modest but statistically significant correlation (*r* = 0.53 *p* = 0.041). Similar analyses performed using iNap, Perceval, and Peredox revealed modest negative correlations (Supplementary Fig. 2). Given NAPDH/NADP+, NADH/NAD+, and ATP/ADP ratios are often associated with the activation of cellular stress response mechanisms, these overall trends may signify that more resistant cell lines compensate more effectively for carboplatin-induced oxidative stress. This observed association suggests that a reduction in oxidative stress, whether through antioxidant adjuvant therapy or an upregulation of stress-mitigating pathways, may indeed sensitize resistant cancer cells to chemotherapies—perhaps via stress-mediated cytotoxicity. However, this relationship remains speculative, and further studies specifically interrogating the effect of oxidative stress-reducing agents on carboplatin resistance are warranted [26].

### Evaluating HyPer signal in 2D, 3D, and in vivo Intravital imaging contexts

Given the robust nature of HyPer expression, we next considered whether application of the sensor in 3D culture could yield more physiologically relevant, ratiometric measurements of intracellular oxidative stress than 2D cultures when compared to OC cells grown in omentum in vivo. Based on extensive evidence in the literature, the ascites-derived OC cell line, SKOV3, was used to recapitulate key features of omental metastasis modeled in 2D, 3D, and intravital contexts [3, 6, 27].

**Fig. 4:**
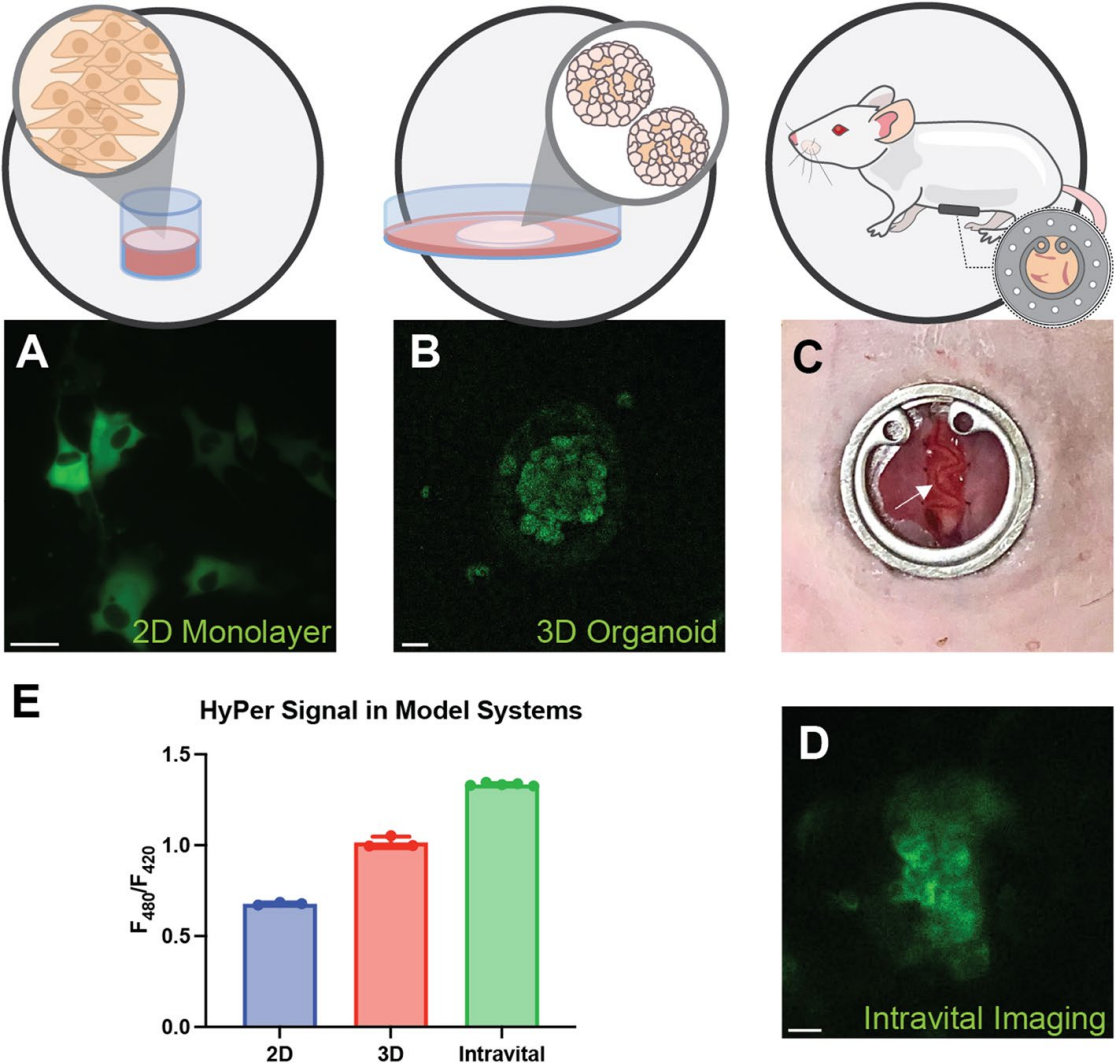
3D organoids more closely recapitulate the oxidative stress of in vivo metastases. SKOV3-Hyper-expressing cells were grown as 2D monolayers or 3D organoids and then injected IP and imaged through an intravital window. Representative fluorescence micrographs of cells imaged in (**A**) 2D and (**B**) 3D organoids. **C** An intravital window was positioned against the mouse omentum (indicated by the white arrow), through which (**D**) omental SKOV3-HyPer cells were imaged in the intravital context. **E** HyPer fluorescence was quantified. Scale bars = 100 um

In order to standardize measurements of the sensor in the different experimental contexts, stable expression of the HyPer-DAAO construct was achieved via lentiviral infection of SKOV3 cells. SKOV3-Hyper cells were then grown as 2D monolayers or in 3D organoids. SKOV3 cells grown in 2D were also injected IP and visualized through a surgically implanted omental intravital window. HyPer signal was measured using matched microscopy settings for all three contexts (2D, 3D, and intravital). Calculations of HyPer measurements in different cell ROIs in 2D, 3D, and intravital contexts revealed that HyPer ratios in organoids more closely resembled the HyPer ratios in metastasizing cells in vivo (Fig. 4).

### Carboplatin-induced changes in HyPer signal in patient-derived Organoids (PDOs) predict patient responses

**Fig. 5:**
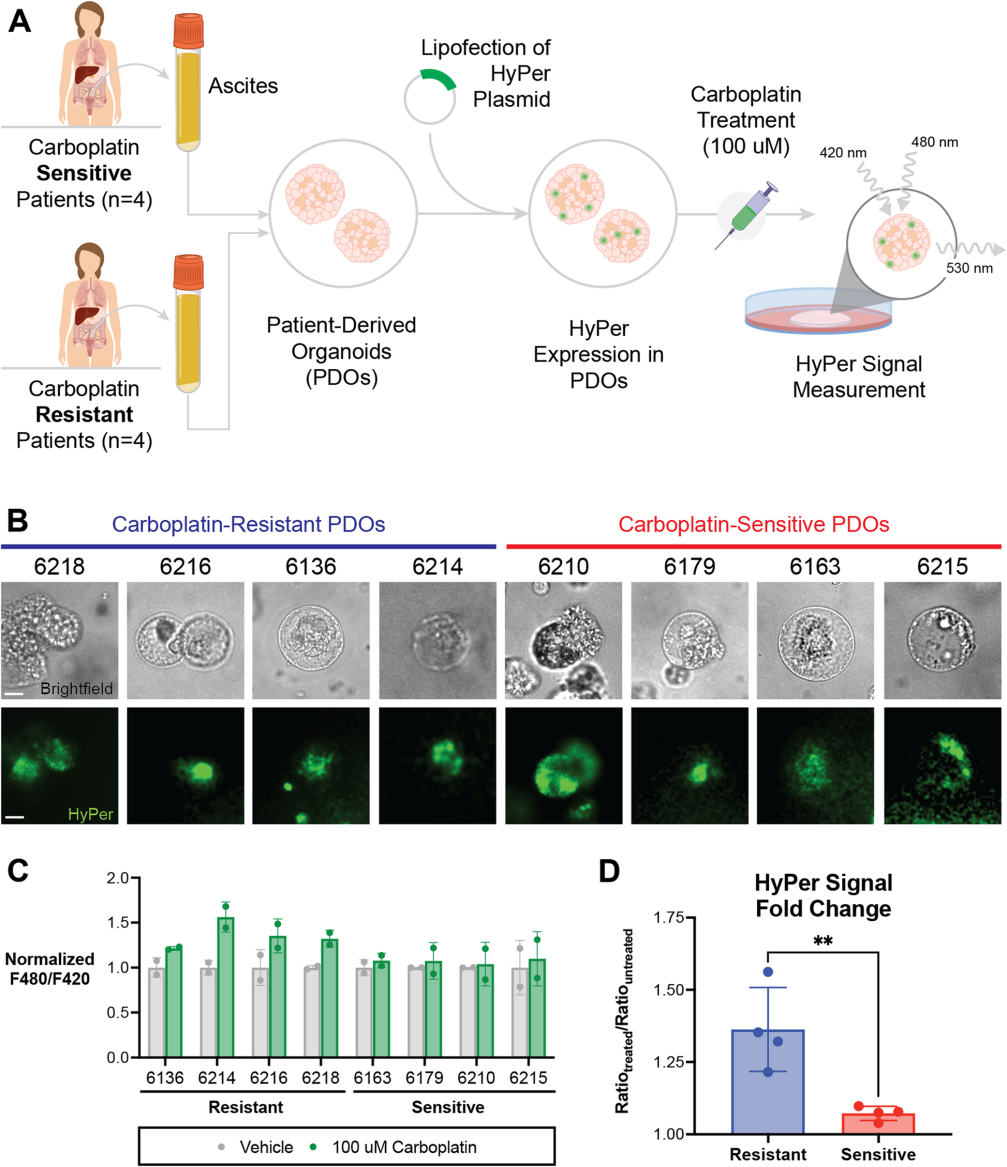
HyPer-expressing PDOs reflect clinical chemoresistance to carboplatin. **A** Schematic of study design. **B** Organoids derived from patient ascites were established and transfected with HyPer, and expression was confirmed using fluorescence microscopy. Scale bars = 200 um. **C** HyPer measurements following carboplatin treatment for 48 hours (*n* = 2 replicates for each patient). **D** The mean fold-change in Hyper signal in PDOs after carboplatin treatment was significantly greater in organoids from carboplatin-resistant patients (*p* < 0.01)

Given our observation that organoid OC oxidative stress more closely resembles in vivo oxidative stress of OC cells, we investigated whether patient-derived organoids (PDOs) expressing HyPer could effectively report metabolic changes, specifically in the context of chemoresistance. A schematic of the study design is shown in Fig. 5A.

We first established organoids from OC cells within malignant ascites taken from carboplatin-resistant (*n* = 4) and carboplatin-sensitive patients (*n* = 4). After plating in Matrigel, cells were transfected with the HyPer-DAAO construct using Lipofectamine. Efficiency of establishing organoids from ascites was 100%, with transfection efficiencies ranging from 20 to 40% in PDOs (Fig. 5B). This relatively low expression was in line with efficiencies reported previously for organoid cultures [28, 29].

HyPer-expressing PDOs were then treated with 100 μM carboplatin for 48 hours, at which time HyPer signals were quantified. As 100 μM is a relatively low dose for organoid culture, significant impacts on cell viability were not noted. HyPer signal in PDOs derived from carboplatin-resistant patients was significantly higher than in PDOs from carboplatin-sensitive patients (Fig. 5C and D).

## Discussion

In recent years, changes in the metabolic landscape of OC have been recognized as important drivers of the two major obstacles to effective treatment of OC patients: metastasis and chemoresistance. Thus, a deeper understanding of the cellular mechanisms that underlie these phenomena is warranted. Organoid models have presented a major advance to understanding OC pathobiology, providing an in vitro tool that effectively recapitulates OC cell and tumor characteristics. However, studying metabolism in these organoid models remains technically challenging and therefore largely unexplored. While cold reagents are applied to quench enzymatic reactions when isolating lysate for metabolomics and enzymatic cycling assays in 2D experiments, lysate isolation from 3D cultures requires matrix digestion, during which metabolic changes may occur [30, 31]. Assays that measure tertiary metabolic readouts like oxygen consumption rate (OCR) or extracellular acidification rate (ECAR) are similarly challenging to apply to organoid cultures, as the diffusion of perturbative reagents through the extracellular matrix required for organoid growth remains highly variable.

Genetically encoded, fluorescent metabolic biosensors present a potential opportunity to study OC metabolism in a robust manner. In this study, we investigated metabolic changes in 15 OC cell lines expressing four metabolic biosensors, iNap, Peredox, Perceval, and HyPer, to measure NADPH/NADP+, NADH/NAD+, ATP/ADP, and intracellular H_2_O_2_ respectively. These sensors use competitive binding of metabolites to active sites as described in Table 1 to provide valuable, dynamic measurements of bioenergetic states. HyPer, Perceval, and iNap are ratiometric sensors, while Peredox includes a conjugated mCherry for normalization. The specific design of these sensors enables measurement of metabolic change while filtering out noise and variation in the levels of biosensor expression in different sample populations. Namely, with the fluorescence at one metabolite saturation inversely proportional to the fluorescence at another, ratiometric calculations allow robust quantification of relative, metabolic states across model systems. While other genetically encoded, metabolic biosensors have been described and are being developed [17, 32], this study serves as a preliminary investigation into the feasibility of using these sensors to explore the metabolic dynamics in OC cells.

Application of these sensors in OC cells revealed changes in cellular metabolism when cells were exposed to OCM, which serves as a surrogate for the omental microenvironment. The OCM-induced increases in intracellular oxidative stress measured using HyPer are particularly interesting in this context, as the precise mechanisms that underlie aggressive metastatic growth in the omentum are not well understood. Integrating this observation with heterogenous NADPH/NADP+ and NADH/NAD+ responses of cell lines may suggest that cellular antioxidant mechanisms are differentially activated to allow cells to alter their metabolism in response to the omental microenvironment. Indeed, recent evidence has also highlighted omental adipocytes as important players dictating the metastatic seeding of OC cells and their eventual reliance on lipid metabolism, suggesting that the simulated OCM microenvironment may only capture a portion of this metabolic transition [3, 6]. Further investigations using metabolic biosensors in both populations—OC cells and omental adipocytes—may yield important insights into the crosstalk that enables aggressive omental metastasis.

Another common clinical challenge in the treatment of patients with OC remains the development of chemoresistance, particularly in response to first-line platinum-based therapeutics. Understanding the role of metabolic machinery in promoting carboplatin resistance may similarly yield insights to guide therapeutic approaches. Unsurprisingly, treatment with carboplatin induced alterations in OC metabolism, including increased oxidative stress and generally, increased NADPH/NADP+ ratios. Interestingly, as a general trend, the degree to which oxidative stress increased appeared to be associated with the carboplatin resistance of the OC cell line. In addition, carboplatin- induced metabolic changes were highly heterogeneous across OC cell lines, likely due to a confluence of intrinsic cellular characteristics including source of cells (primary tumors vs. ascites), maintenance methods, and patient factors.

In recent years, the role of metabolism as a driver of cellular behaviors and clinical outcomes has been increasingly appreciated, underscoring a critical need to identify which models can adequately reproduce in vivo metabolic behaviors in a robust way. While 2D cell lines have offered a low cost, high throughput model, a growing body of evidence suggests that novel 3D models—in particular organoid cultures—may offer a more faithful representation of in vivo physiology [33, 34]. While these newer culture methods have drawbacks as well (challenging reproducibility, increased technical expertise, access to specialized reagents, etc.), the advantages they offer over high-cost, low-throughput murine models remain of interest. Thus, further studies to fully understand the applicability and requirements for using these organoid models in studying metabolism are necessary.

In this preliminary effort to understand if these observed changes could be recapitulated in 3D organoids, we established 3D organoids from SKOV3-HyPer expressing cells. We also injected SKOV3-HyPer expressing cells intraperitoneally and imaged omental seeding of these cells through an intravital window. Performing live imaging in these three contexts (2D, 3D, and in vivo) allowed us to determine which most closely modeled in vivo metabolism. Organoids appeared to be a robust model for in vivo metabolism, more closely reflecting the measured oxidative stress of in vivo OC cells compared to 2D monolayers.

Perhaps more importantly, the strength of the organoid approach for modeling OC cell metabolism in vivo allowed us to investigate the relationship between OC carboplatin resistance and oxidative stress in patient-derived samples. Indeed, we found using this approach that in PDOs established from OC patients, the oxidative stress measured in organoids derived from carboplatin-resistant patients was significantly higher than that measured in organoids from carboplatin-sensitive patients following carboplatin treatment.

The observation of greater oxidative stress in OC cell lines and in OC PDOs could be important for our understanding of the mechanisms of OC, however further studies are necessary to fully elucidate the mechanistic underpinnings of this finding. It is interesting to speculate that activation of oxidative stress pathways could activate cellular response mechanisms that may select for resistant cells. Indeed, a recent study that used longitudinal single-cell RNA-seq to analyze metastatic OC patient tumors revealed that therapy-induced stress clonally selected for resistant phenotypes [35]. The temporality of this relationship also remains unclear, as it is possible that a chronic reliance on an altered metabolic pathway may result in more permanent metabolic vulnerability. Specific targeting of metabolism during the window of downregulation of particular metabolic enzymes and upregulation of others may present a therapeutic opportunity, which should be the subject of further studies in the future. Taken together, the observed relationship between oxidative stress and carboplatin-resistant OC may provide an opportunity for synergistic therapy with oxidative stress-reducing agents that could sensitize resistant cells to carboplatin.

Our work represents early pre-clinical work to establish the validity of using biosensors as tools to interrogate OC metabolism. Further investigations are necessary to expand the scope of the conclusions of this study. For example, it remains unclear how faithfully organoids recapitulate in vivo tumor dynamics. Although the sensors used here provided valuable information, an important limitation is that they compete with endogenous enzymes for intracellular metabolites, thereby potentially altering the cellular processes they are intended to measure. Although previous studies have suggested that this effect is minimal, it requires ongoing consideration [19, 21]. Moreover, appropriate controls using exogenous stimuli to establish biosensors’ responses are necessary.

Finally, while this initial analysis of OC organoids derived from carboplatin-resistant and carboplatin-sensitive patients presents a promising proof-of-concept, the sample size was small (*n* = 4 per group). The high rates of establishment for ascites-derived OC organoids suggests ascites may be a robust source material. In clinical settings, ascites is often drained for symptomatic management and discarded. Further studies using larger sample sizes from more heterogenous patient populations may expand our understanding of the predictive capability of biosensor signals. Despite these limitations, this study illustrates that genetically encoded, fluorescent biosensors can be used to study OC organoids, presenting an unprecedented opportunity to study OC metabolism and pathobiology.

## Conclusions

Genetically encoded, fluorescent biosensors are valuable tools for studying the evolving field of OC metabolism. As novel methods for 3D organoid cultures become more common, the application of these tools will likely prove to be even more essential to growing our understanding of OC pathobiology. Specifically, investigations using these tools may yield important insights to overcome chemoresistance and metastasis that prevent durable remissions and response to treatment.

## Supplementary Information

**Additional file 1: Supplementary Figure 1.** OC cell lines exhibit heterogenous biosensor responses to OCM treatment. Fluorimetric measurements of iNap (A), Perceval (B), and Peredox (C) signal were normalized to the average across all cell lines (*n* = 3 technical replicates per cell line). Signal was measured following 48 hours of growth in OCM or regular media and normalized to growth in control regular media for iNap (D), Perceval (E), and Peredox (F).

**Additional file 2: Supplementary Figure 2.** Biosensor responses to carboplatin treatment across heterogenous OC cell lines are negatively correlated with carboplatin resistance. Biosensor-expressing cell lines were treated with 100 μM carboplatin and iNap (A), Perceval (B), and Peredox (C) signal was measured at 24 and 48 hours. (D-F) Signal for each biosensor in (A-C) in treated cells was compared to that in control, untreated cells, normalized to 1 (*n* = 3 per condition). Normalized iNap (G), Perceval (H), and Peredox (I) responses were negatively correlated with reported carboplatin IC50s for each cell line (Supplementary Table 1).

**Additional file 3: Supplementary Table 1.** Carboplatin IC50s for OC Cell Line Screen. Adapted from E Bicaku et al. 2012 and Krietzburg et al. 2019.

**Additional file 4: Supplementary Table 2.** OC Organoid Media Composition.
