# Supplementary figures and images for: Using genetically encoded fluorescent biosensors to interrogate ovarian cancer metabolism
Source: J Ovarian Res. 2022 Oct 20;15:114. doi: 10.1186/s13048-022-01046-5 (PMC9585869; doi:10.1186/s13048-022-01046-5)

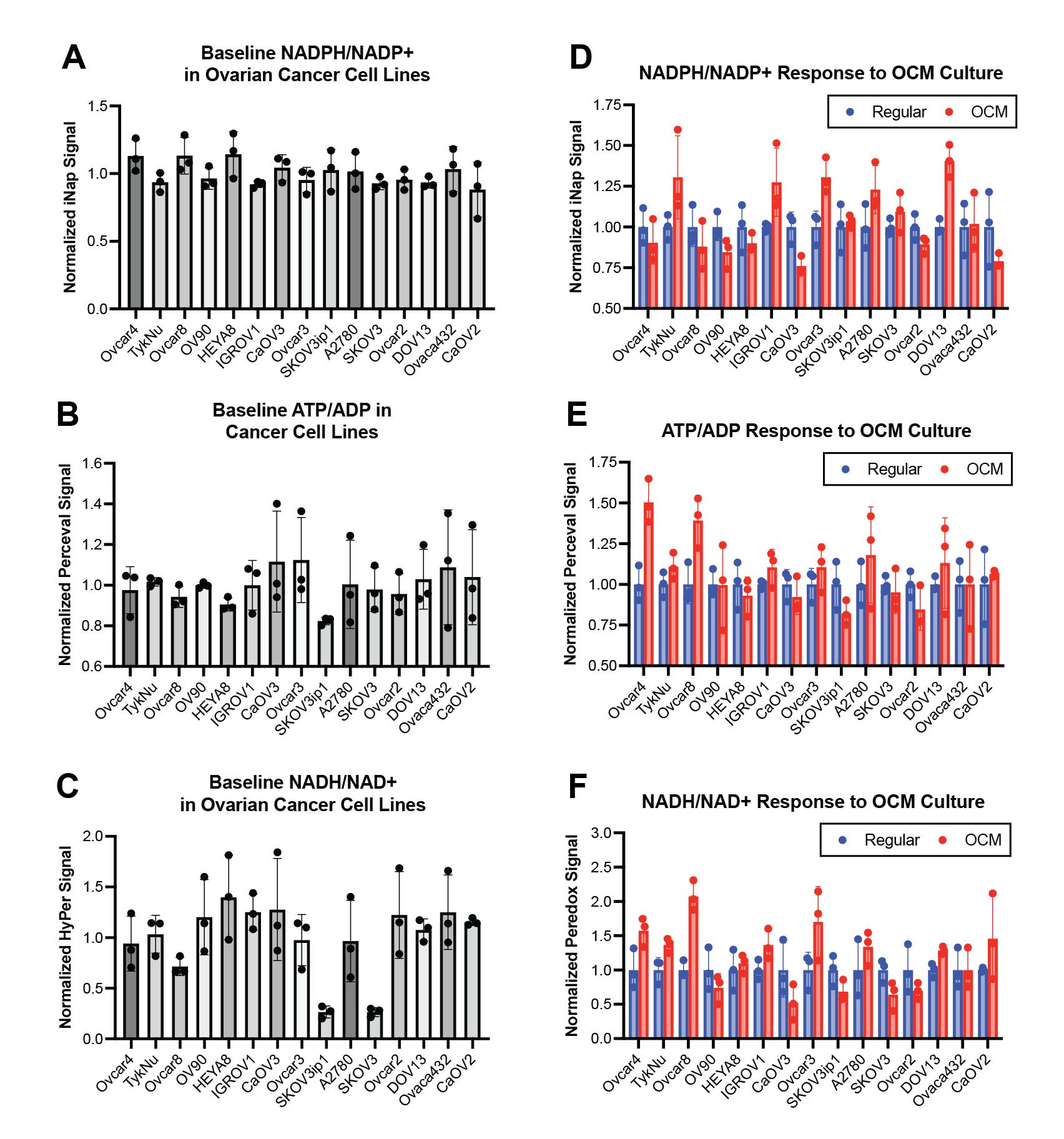

Supplement: Supplementary file 1 — Additional file 1: Supplementary Figure 1. OC cell lines exhibit heterogenous biosensor responses to OCM treatment. Fluorimetric measurements of iNap (A), Perceval (B), and Peredox (C) signal were normalized to the average across all cell lines (n = 3 technical replicates per cell line). Signal was measured following 48 hours of growth in OCM or regular media and normalized to growth in control regular media for iNap (D), Perceval (E), and Peredox (F). [file 13048_2022_1046_MOESM1_ESM.png]

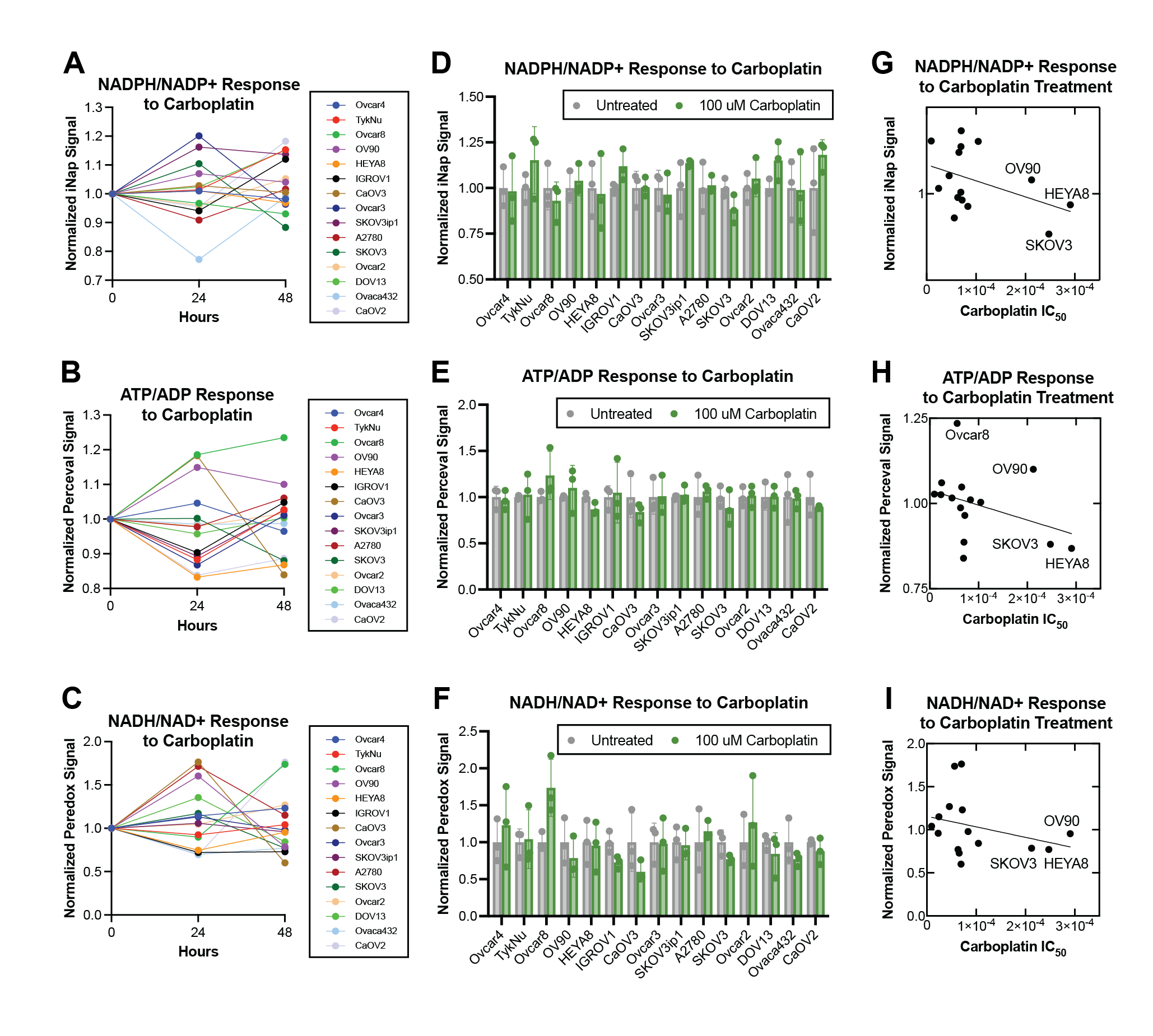

Supplement: Supplementary file 2 — Additional file 2: Supplementary Figure 2. Biosensor responses to carboplatin treatment across heterogenous OC cell lines are negatively correlated with carboplatin resistance. Biosensor-expressing cell lines were treated with 100 μM carboplatin and iNap (A), Perceval (B), and Peredox (C) signal was measured at 24 and 48 hours. (D-F) Signal for each biosensor in (A-C) in treated cells was compared to that in control, untreated cells, normalized to 1 (n = 3 per condition). Normalized iNap (G), Perceval (H), and Peredox (I) responses were negatively correlated with reported carboplatin IC50s for each cell line (Supplementary Table 1). [file 13048_2022_1046_MOESM2_ESM.png]
