# Supplementary material for: Using genetically encoded fluorescent biosensors to interrogate ovarian cancer metabolism
Source: J Ovarian Res. 2022 Oct 20;15:114. doi: 10.1186/s13048-022-01046-5 (PMC9585869; doi:10.1186/s13048-022-01046-5)
Supplement: Supplementary file 3 — Additional file 3: Supplementary Table 1. Carboplatin IC50s for OC Cell Line Screen. Adapted from E Bicaku et al. 2012 and Krietzburg et al. 2019. [file 13048_2022_1046_MOESM3_ESM.docx]

|  | **Carboplatin Treatment** | | | |
| --- | --- | --- | --- | --- |
| **Cell line** | **IC50 mean** **(M)** | **n** | **Mean Standard Error (SEM)** | **Relative Standard Error (RSE)** |
| **A2780** | 24.3E-6 | 14 | 7.2E-6 | 29.7 |
| **CAOV2** | 70.2E-6 | 19 | 15.7E-6 | 22.4 |
| **CAOV3** | 69.4E-6 | 5 | 18.0E-6 | 25.9 |
| **Dov13** | 104.5E-6 | 10 | 30.6E-6 | 29.3 |
| **HeyA8** | 290.8E-6 | 8 | 34.5E-6 | 11.9 |
| **IGROV1** | 65.4E-6 | 3 | 26.2E-6 | 40.0 |
| **OV90** | 212.6E-6 | 7 | 27.1E-6 | 12.7 |
| **OVCA432** | 63.1E-6 | 5 | 5.2E-6 | 8.3 |
| **OVCAR2** | 45.5E-6 | 11 | 5.0E-6 | 11.0 |
| **OVCAR3** | 83.7E-6 | 4 | 33.1E-6 | 39.5 |
| **OVCAR4** | 72.0E-6 | 22 | 8.3E-6 | 11.6 |
| **OVCAR8** | 56.1E-6 | 5 | 2.3E-6 | 4.1 |
| **SKOV3** | 247.4E-6 | 8 | 29.8E-6 | 12.0 |
| **SKOV3ip1** | 23.0E-6 | 11 | 3.2E-6 | 13.8 |
| **Tyknu** | 9.6E-6 | 7 | 1.8E-6 | 18.6 |

**Supplementary Table 1: Carboplatin IC50s for OC Cell Line Screen.** Adapted from E Bicaku et al. 2012 and Krietzburg et al. 2019.
