# Supplementary material for: Using genetically encoded fluorescent biosensors to interrogate ovarian cancer metabolism
Source: J Ovarian Res. 2022 Oct 20;15:114. doi: 10.1186/s13048-022-01046-5 (PMC9585869; doi:10.1186/s13048-022-01046-5)
Supplement: Supplementary file 4 — Additional file 4: Supplementary Table 2. OC Organoid Media Composition. [file 13048_2022_1046_MOESM4_ESM.docx]

| Media Component | Final Concentration |
| --- | --- |
| DMEM/F12 |  |
| GlutaMax | 1x |
| Penicillin/Streptomycin | 100 U/mL |
| 17-B-Estradiol | 10 nM |
| A083-01 | 250 nM |
| B27 (without Vitamin A) | 1x |
| EGF | 50 ng/mL |
| HGF | 10 ng/mL |
| IGF1 | 20 ng/mL |
| N2 Supplement | 1x |
| N-Acetylcysteine | 5 mM |
| Neuregulin I | 10 ng/mL |
| Nicotinamide | 5 mM |
| Noggin | 100 ng/mL |
| R-Spondin 1 | 50 ng/mL |
| SB203580 (p38i) | 1 uM |
| Y-27632 | 10 uM |

**Supplementary Table 2:** OC Organoid Media Composition
